# A Monoclonal Antibody against p53 Cross-Reacts with Processing Bodies

**DOI:** 10.1371/journal.pone.0036447

**Published:** 2012-05-10

**Authors:** María Gabriela Thomas, Luciana Luchelli, Malena Pascual, Vanesa Gottifredi, Graciela Lidia Boccaccio

**Affiliations:** 1 Instituto Leloir Av. Patricias Argentinas Buenos Aires, Argentina; 2 IIBBA-CONICET, Buenos Aires, Argentina; 3 Facultad de Ciencias Exactas y Naturales, University of Buenos Aires, Argentina; International Centre for Genetic Engineering and Biotechnology, Italy

## Abstract

The p53 tumor suppressor protein is an important regulator of cell proliferation and apoptosis. p53 can be found in the nucleus and in the cytosol, and the subcellular location is key to control p53 function. In this work, we found that a widely used monoclonal antibody against p53, termed Pab 1801 (Pan antibody 1801) yields a remarkable punctate signal in the cytoplasm of several cell lines of human origin. Surprisingly, these puncta were also observed in two independent p53-null cell lines. Moreover, the *foci* stained with the Pab 1801 were present in rat cells, although Pab 1801 recognizes an epitope that is not conserved in rodent p53. In contrast, the Pab 1801 nuclear staining corresponded to genuine p53, as it was upregulated by p53-stimulating drugs and absent in p53-null cells. We identified the Pab 1801 cytoplasmic puncta as P Bodies (PBs), which are involved in mRNA regulation. We found that, in several cell lines, including U2OS, WI38, SK-N-SH and HCT116, the Pab 1801 puncta strictly colocalize with PBs identified with specific antibodies against the PB components Hedls, Dcp1a, Xrn1 or Rck/p54. PBs are highly dynamic and accordingly, the Pab 1801 puncta vanished when PBs dissolved upon treatment with cycloheximide, a drug that causes polysome stabilization and PB disruption. In addition, the knockdown of specific PB components that affect PB integrity simultaneously caused PB dissolution and the disappearance of the Pab 1801 puncta. Our results reveal a strong cross-reactivity of the Pab 1801 with unknown PB component(s). This was observed upon distinct immunostaining protocols, thus meaning a major limitation on the use of this antibody for p53 imaging in the cytoplasm of most cell types of human or rodent origin.

## Introduction

The p53 tumor suppressor is a key factor involved in the cellular response to the accumulation of damaged DNA and other cell insults like hypoxia, oncogene expression, nutrient deprivation and ribosome dysfunction [1]. p53 transactivates a number of genes with a variety of functions including cell cycle arrest, apoptosis and metabolism regulation, among others [1]. In addition, p53 has transcription-independent functions that depend on its localization in the cytoplasm, where p53 modulates apoptosis and autophagy [2]. While the pro-apoptotic role of cytoplasmic p53 was linked to its recruitment to the mitochondria [2], [3], several groups have shown that cytoplasmic p53 is not limited to this organelle. Strong cytoplasmic p53 retention was reported in neuroblastoma cells and other cell lines, including human fibroblasts upon endoplasmic reticulum stress [4], [5], [6], [7], [8], [9], [10]. A number of specific proteins that interact with p53 in the cytoplasm precluding its nuclear import and thus neutralizing p53-dependent transcriptional activation were described by several groups [4], [5], [6], [7], [8], [9], [10]. In line with this, discrete p53 cytoplasmic aggregates that may represent sites for p53 storage were described under a variety of conditions [5], [6], [7], [9].

In this work, we used several antibodies to visualize the subcellular distribution of p53 in several cell lines exposed to different stimuli. We found that a particular monoclonal antibody, termed Pantropic antibody 1801 (Pab 1801), yields a strongly punctate signal in the cytoplasm of several human cell lines. Strikingly, these *foci* are also present in p53-negative cells and in rat cells, which lack the p53 epitope that is specifically recognized by the Pab 1801. Further analysis clearly indicated that the Pab 1801-positive *foci* colocalize with P bodies, which are conserved cytoplasmic aggregates of RNPs involved in mRNA storage, silencing and/or decay (reviewed in ref. [11]). PBs are dynamic and we found that the Pab 1801 punctate signal vanishes upon PB dissolution. Our results unveil a strong cross-reactivity of the Pab 1801 with PB components, upon a variety of staining conditions, thus indicating a major limitation of this widely used antibody for p53 imaging in most cell types.

## Results

### The Pab 1801 Against p53 Yields a Granular Cytoplasmic Signal

**Figure 1:**
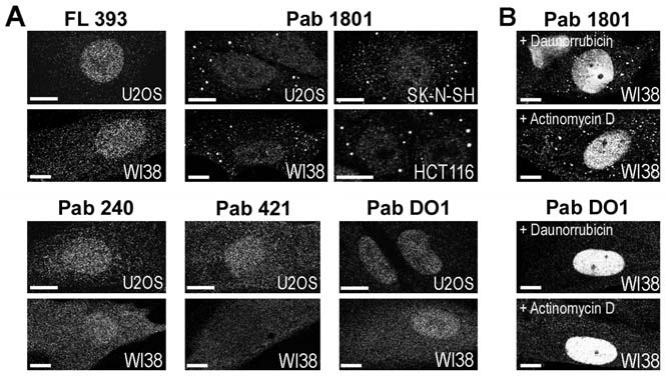
The Pab 1801 yields a cytoplasmic punctate pattern. U2OS, WI38, SK-N-SH, and HCT116 cells were fixed in PFA, permeabilized in Triton 0.1% and immunostained with the indicated antibodies against p53 as indicated in Materials and Methods. A, Under resting conditions, the Pab 1801 showed a cytoplasmic granular pattern, whereas the FL 393, the Pab 240, the Pab 421 and the Pab DO1 antibodies yielded a faint nuclear signal. B, WI38 cells were treated with daunorubicin or actinomycin D, and immunostained with the indicated antibodies. A strong p53 nuclear signal was observed in all cases. The Pab 1801 punctate signal in the cytoplasm remained unaltered. Bar, 10 µm.

We immunostained distinct cell lines, specifically U2OS, WI38, SK-N-SH and HCT116, with a rabbit polyclonal antibody termed FL 393, and with a number of monoclonal antibodies against p53, namely Pab 1801, Pab 240, Pab 421 and Pab DO1 (see materials and methods), all them widely used in the literature [6], [7], [8], [9], [12], [13], [14]. Cells were fixed in PFA 4%, permeabilized and stained as indicated in material and methods. In all the cell lines tested, the Pab 1801 showed a punctate pattern with granules of about 0.5 µm in diameter, quite homogenously dispersed in the cytoplasm (Figure 1A). This staining pattern is quite similar to that previously described by Moll et al. in SK-N-SH neuroblastoma cells using a different staining procedure that includes non-aqueous fixation [6], [7]. The remaining antibodies against p53 display the faint nuclear staining typically observed under resting conditions, and showed no significant signal in the cytosol (Figure 1 A and B).

To investigate whether the distinct pattern of the Pab 1801 is affected by increased p53 levels, we induce p53 in WI38 cells with daunorubicin, a DNA- damaging drug, or with actinomycin D, which does not induce DNA-damage but nevertheless causes p53 accumulation [15], and stained the cells with the Pab 1801 or the Pab DO1. As expected, we found that the p53 nuclear signal increased in both cases (Figure 1B and data not shown). In contrast, the cytoplasmic puncta stained by the Pab 1801 remained unaltered. They did not change in number (between 8 and 12 per cell in all cases), size (about 0,5 µm in all conditions) or brightness (Figure 1B and data not shown), indicating that these structures are not affected when p53 levels are upregulated. Pab DO1 staining was exclusively nuclear upon drug treatment, and no cytosolic signal was observed despite the dramatic increase on p53 levels (Figure 1 B).

### The Decay of p53 Upon Translation Inhibition does not Correlate with a Decay of the Cytoplasmic Puncta

**Figure 2:**
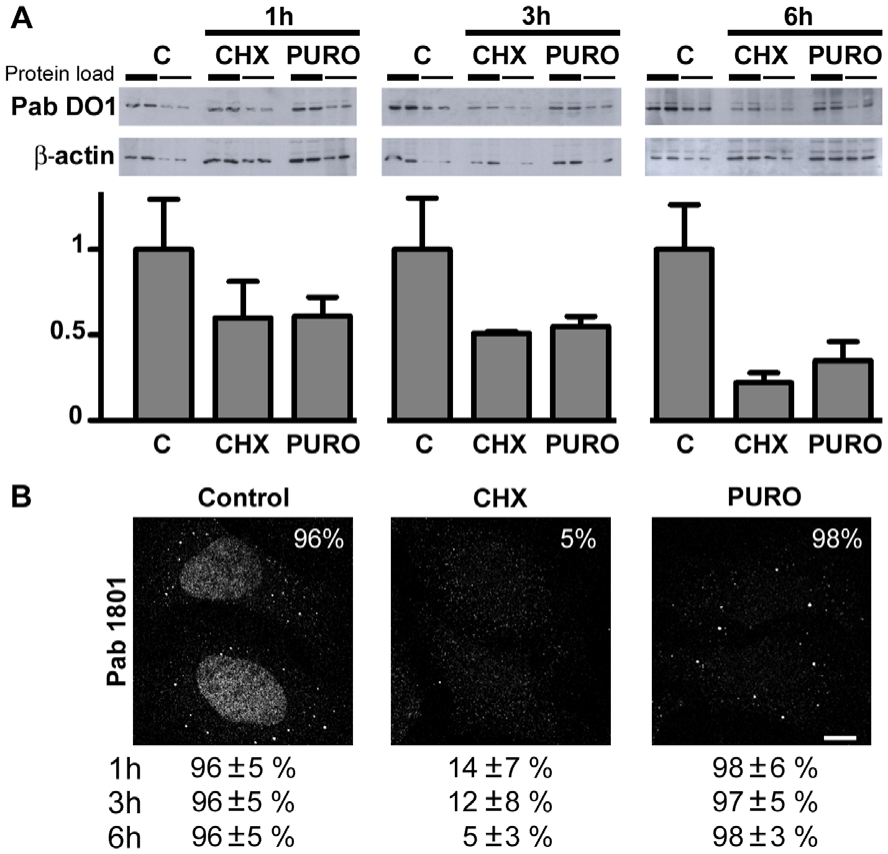
p53 decay upon translation inhibition does not correlate with Pab 1801 puncta disappearance. U2OS cells were treated with cycloheximide (CHX) or puromycin (PURO) during 1, 3, or 6 hs. C, control. A, western blot with the Pab DO1. Duplicates of two-fold dilutions from each treatment were loaded. p53 levels were determined and normalized using beta-actin as loading control. Error bars, standard deviation. p53 decay was comparable upon protein synthesis inhibition by cycloheximide or puromycin. B, Cells were stained with the Pab 1801. Two representative cells upon 6 hs-treatment are shown. The percentage of cells with punctate Pab 1801 signal was evaluated in 100 cells from duplicate stainings for each treatment at the indicated time points. The Pab 1801 puncta vanished upon cycloheximide treatment and remained unaffected upon puromycin exposure. Bar, 10 µm.

p53 decays rapidly upon translation blockage. Then, we sought to investigate whether the 1801-positive puncta decay upon treatment of U2OS cells with two translation inhibitors, cycloheximide (CHX) and puromycin (PURO). In parallel, we evaluated p53 levels by western blotting using the DO1 antibody, one of the most used antibodies for p53 immunoblotting. As expected, we found that p53 decayed rapidly upon inhibition of protein synthesis with cycloheximide or puromycin. In both cases, p53 levels were reduced to 60% relative to basal levels after 1 hour treatment, to half after three hours, and to one third upon 6 hours (Figure 2A). Strikingly, the Pab 1801 cytoplasmic puncta showed a quite distinct behaviour. They vanished upon exposure to cycloheximide, but were unaffected by puromycin. The proportion of cells with visible Pab 1801 puncta were reduced from 96% to 5% upon 6 h of cycloheximide treatment, but remained high (98%) upon exposure to puromycin (Figure 2B), although in both cases p53 levels were significantly reduced. This strongly suggests that in addition to nuclear p53, the Pab 1801 recognizes cellular components distinct from p53 and present in cytoplasmic *foci*.

### The Pab 1801-puncta are Present in p53-negative Cells

**Figure 3:**
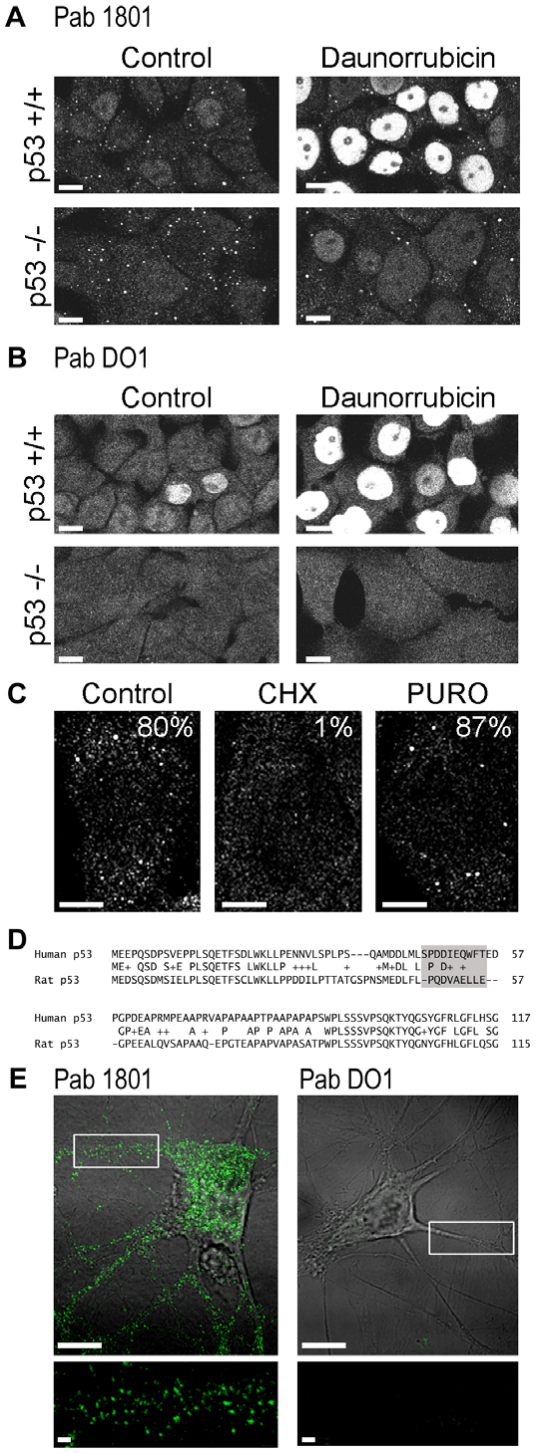
Pab 1801 puncta are present in p53-null cells. A, B, p53+/+ or p53−/− HCT116 cells were treated with daunorubicin and immunostained with the Pab 1801 (A) or the Pab DO1 (B). With both antibodies, a nuclear signal is observed upon stimulation of p53+/+ cells but not p53−/− cells. Pab 1801-positive puncta are always present in p53+/+ and p53−/− cells independently of p53 levels. C, p53-null H1299 cells were treated with the indicated drugs and stained with the Pab 1801. The Pab 1801 puncta disappeared upon exposure to cycloheximide and remained unaltered after puromycin treatment. The percentage of cells with punctate Pab 1801 signal was evaluated in 100 cells from duplicate stainings for each treatment. Bar, 10 µm. D, alignment of human and rat p53 N-terminus including the Pab 1801 epitope (grey box). The 10-aa epitope is absent in the rat sequence. E, Hippocampal rat neurons were prepared as described [27] and stained with the Pab 1801 or the Pab DO1. A merge of the immunofluorescence and the phase contrast images is shown. Pab 1801-positive puncta are detected in cell soma and dendrites. Bars, whole cells, 10 µm; magnifications, 1 µm.

Then, we assessed the Pab 1801 staining in p53-negative cell lines. We used p53−/− HCT116 cells, where both p53 alleles are disrupted by homologous recombination [16]. Strikingly, we found that the Pab 1801 recognized cytoplasmic puncta, as in p53+/+ HCT116 cells (Figure 3A), that were similar to those present in other cell lines (see Figure 1A). To ascertain that these cells lack p53, we exposed p53+/+ and p53−/− HCT116 cells to daunorubicin. As expected, we found that the p53 nuclear signal increased in p53+/+ HCT116 but not in p53−/− cells (Figure 3A). As above, the cytoplasmic puncta stained by the Pab 1801 remained unaltered upon DNA damage induction (Figure 3A). Finally, the DO1 antibody did not detect any cytosolic puncta in these cells, exposed or not to DNA damage agents, which upregulated p53 strongly (Figure 3B). The Pab 1801 also stained cytoplasmic *foci* in p53-negative H1299 cells, which have a homozygous partial deletion of the p53 gene that precludes p53 expression (Figure 3C) ([17]. Also see detailed description in ATCC CRL-5803 web page (http://www.atcc.org/ATCCAdvancedCatalogSearch/ProductDetails/tabid/452/Default.aspx?ATCCNum=CRL-5803&Template=cellBiology). Moreover, as described for p53-positive cells (see Figure 2B), the Pab 1801 puncta detected in p53-negative H1299 cells dissolved upon treatment with cycloheximide, and remained unaltered upon exposure to puromycin (Figure 3C). Altogether, these observations indicate that the Pab 1801 cross-react with cytoplasmic structures that apparently do not contain p53.

The Pab 1801 recognizes an epitope at the N-terminal region of human p53 and does not recognize rodent p53 [18], [19], as the amino acid sequence is not conserved (Figure 3D). Then, we sought to investigate whether the Pab 1801-positive cytoplasmic puncta were as well present in rat cells. We stained primary cultures of rat hippocampal neurons with the Pab 1801 and found numerous *foci* in the soma and dendrites (Figure 3E). As in human cell lines (Figures 1A, 1B, 3A, 3B), the DO1 antibody did not stain any cytoplasmic puncta in rat neurons. Collectively, these observations indicate that in addition to p53, the Pab 1801 recognizes an unknown cellular component that is conserved in human and rat cells.

### The Pab 1801 Puncta Colocalize with PBs

**Figure 4:**
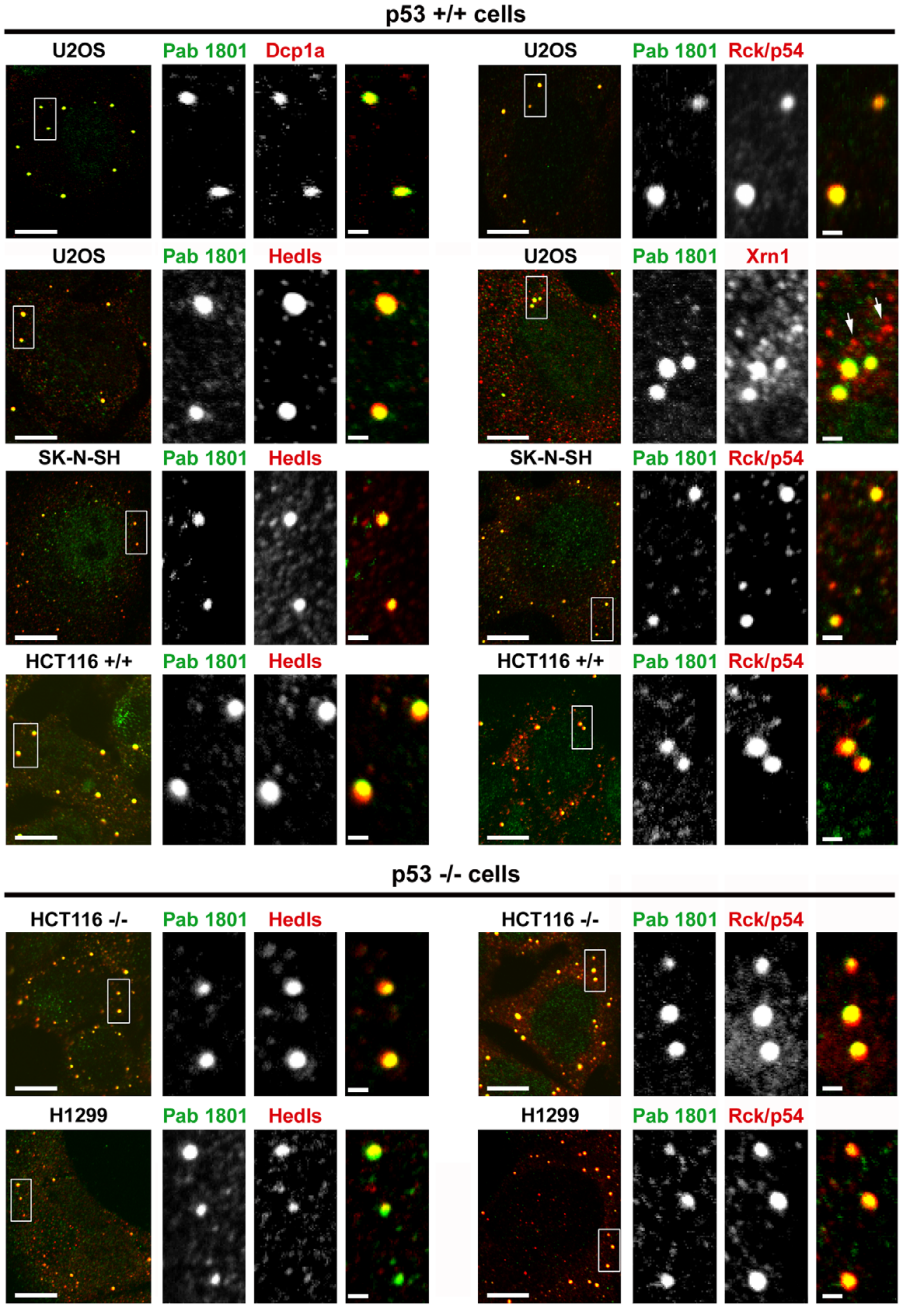
The Pab 1801-positive puncta contain PB components. The indicated p53+/+ or −/− cell lines were simultaneously immunostained with Pab 1801 and antibodies specific for the PB components Dcp1a, Rck/p54, Hedls or Xrn1. In all cases, all the cytoplasmic *foci* recognized by the Pab 1801 were also recognized by the antibodies against PB markers. Bars, whole cells, 10 µm; magnifications, 1 µm.

**Figure 5:**
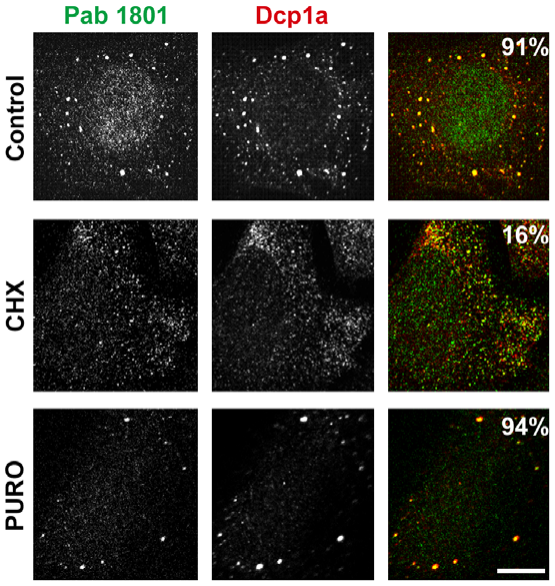
PBs and Pab 1801-positive puncta simultaneously dissolve upon polysome stabilization. U2OS cells were treated with CHX or PURO during one hour, and immunostained with the indicated antibodies. The Pab 1801 punctate signal vanished together with the PBs upon cycloheximide treatment. Both *foci* remained intact upon exposure to puromycin. The percentage of cells with PBs simultaneous stained with Dcp1a and Pab 1801 was evaluated in 100 cells from duplicate experiments for each treatment. The Pab 1801 *foci* were never observed free of Dcp1a, and PBs were always stained with the Pab 1801. Bar, 10 µm.

The differential sensitivity of the Pab 1801 puncta to cycloheximide or puromycin, two strong translational inhibitors, suggests that they are related to mRNA silencing *foci*. Briefly, mRNA silencing *foci* are cytoplasmic accretions of silenced mRNPs that exchange mRNA with the cytosol and translating polysomes. The so-called processing bodies (PBs) and stress granules (SGs) are the two main silencing *foci* described to date. Both PBs and SGs dissolve when polysomes are stabilized by cycloheximide, and remain unaffected or enhanced when polysomes are disrupted by puromycin, as a consequence of the rapid shuttling of mRNAs between the polysomes and the silencing *foci* (reviewed in [11], [20], [21]). Thus, the dissolution of Pab 1801 puncta upon polysome stabilization by cycloheximide, and their resistance to polysome breakdown by puromycin correlate with the behaviour of PBs and SGs. Then, we sought to investigate whether the Pab 1801-stained puncta colocalize with these mRNA silencing *foci*. First, we simultaneously stained p53+/+ or p53−/− cells with the Pab 1801 and commercial antibodies that specifically recognize a number of accepted PB markers: decapping enzyme 1a (Dcp1a), Rck/p54, Hedls and exoribonuclease enzyme 1 (Xrn1). We found that in all cases, 100% of the Pab 1801-positive puncta contained all the PB components tested (Figure 4). Conversely, all the PBs identified by Dcp1a, Rck/p54 or Hedls were also recognized by the Pab 1801 (Figure 4). The Xrn1 *foci* showed a slightly different behaviour, and the smallest Xrn1 *foci* (arrows) were not recognized by the Pab 1801, suggesting that this anti-p53 antibody does not cross-react with Xrn1, but rather with some other protein that concentrates in the largest PBs. Additionally supporting the identification of the 1801-puncta as PBs, we found that they show the same response upon polysome stabilization. The Pab 1801 *foci* and the PBs vanished simultaneously when cells were exposed to cycloheximide, whereas translation inhibition by puromycin elicited no effect (Figure 5 and see also figure 2 above).

**Figure 6:**
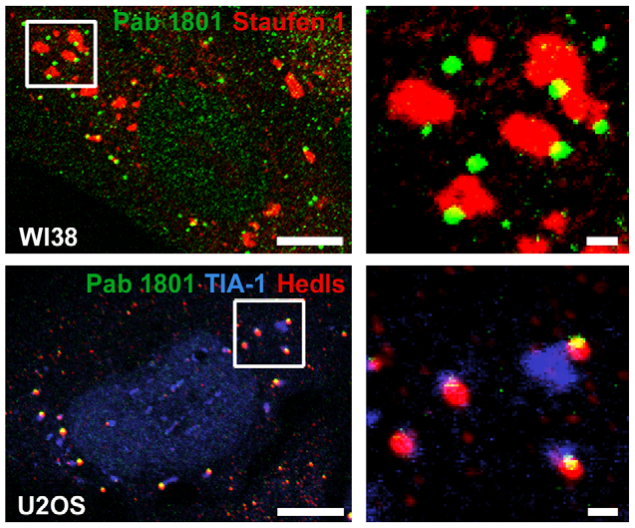
Pab 1801 puncta colocalizes with PBs under stress conditions . WI38 or U2OS cells were treated with 1 µM thapsigargin during one hour, and immunostained with the indicated antibodies. The Pab 1801 puncta do not colocalize with SGs, but are adjacent to them and always overlap with PBs, as in Figures 4 and 5. Bars, whole cells, 10 µm; magnifications, 1 µm.

To investigate whether the Pab 1801 recognizes SGs, we exposed the cells to thapsigargin, a known inductor of ER-stress (Figure 6). SGs were visualized by immunostaining of the SG components Staufen or TIA-1. As expected [22], [23], we found that thapsigargin triggers SG formation in 80 to 100% of the cells. We simultaneously stained these cells with the Pab 1801, and found that in most cases the Pab 1801 puncta were adjacent to the SGs recognized by either Staufen or TIA-1 (Figure 6). This association with SGs is typical of PBs and indeed, simultaneous staining of the PB marker Hedls revealed that the Pab 1801 puncta overlap with PBs also under stress conditions. Similar results were obtained upon SG induction by arsenite treatment, a known inducer of oxidative stress (data not shown).

**Figure 7:**
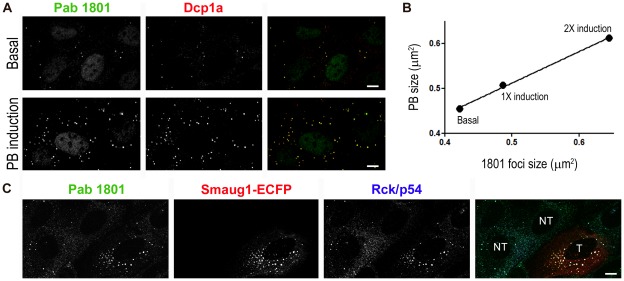
Pab 1801 puncta and PBs are simultaneously induced by different strategies. A, B, U2OS cells were treated with two different combinations of arsenite and puromycin: 250 µM arsenite +100 µg/ml puromycin (1× induction) and 500 µM arsenite +250 µg/ml puromycin (2× induction). A, The average number of PBs per cell increased from 4 in control cells to 14 in treated cells, as evaluated in 100 cells from duplicate experiments for each treatment. Induced PBs were stained with the Pab 1801 in all cases. An example of the 2× induction is shown in Ars+Puro panel. B, the size of Dcp1a-foci and Pab 1801 foci increased at the same rate and in a dose-dependent manner. Median values of more than 100 foci size for each treatment were plotted. C, overexpression of the translational repressor Smaug 1 also provoked 1801 foci induction. U2OS cells were transfected with Smaug 1-ECFP and number of foci per cell and foci size were analized in transfected (T) and neighbouring non-transfected cells (NT). The average number of PBs per cell increased from 5 in non-transfected cells to 55 in transfected cells, as evaluated in 100 hundred cells of each condition. In all cases PBs were stained with the 1801 antibody. The foci size increased more than twice, as evaluated for both 1801 and Rck/p54 in 200 non-transfected foci and 750 Smaug1-ECFP transfected foci. Bars, 10 µm.

To further assess the colocalization of the 1801 puncta with PBs, we sought to induce PB formation. Among several described strategies that enhance the number of PBs like: c-Jun induction by Il-1 treatment [24], RhoA activation by glucose deprivation [25], polysome disassembly [11] and overexpression of translational repressors [26], [27], [28], we chose the last two approaches. First we used different combinations of arsenite and puromycin to gradually enhance PB formation [11]. As expected, we found that the number of PBs per cell and their size increased gradually with the strength of treatment, and induced PBs were stained with the Pab 1801 in all cases (Figure 7A). Untreated cells had an average of 4 Pab 1801 puncta per cell, and this number increased to 14 Pab 1801 puncta per cell upon PB induction. Moreover, the size of both the Dcp1a-*foci* and Pab 1801, which strictly colocalized, increased at the same rate and in a dose-dependend effect (Figure 7B).

Second, we overexpressed a translational repressor, a manipulation known to enhance the number of PBs [26], [27], [28]. We transfect cells with the translational repressor Smaug1, that dramatically induce PBs [27]. We found that the average number of 1801 puncta per cell increased from 5 to 55, and in all cases they were stained with PB markers (Figure 7C). In addition, *foci* size in transfected cells was more than twice that in neighbouring non-transfected cells, as evaluated for both 1801 and Rck/p54 (Figure 7C).

**Figure 8:**
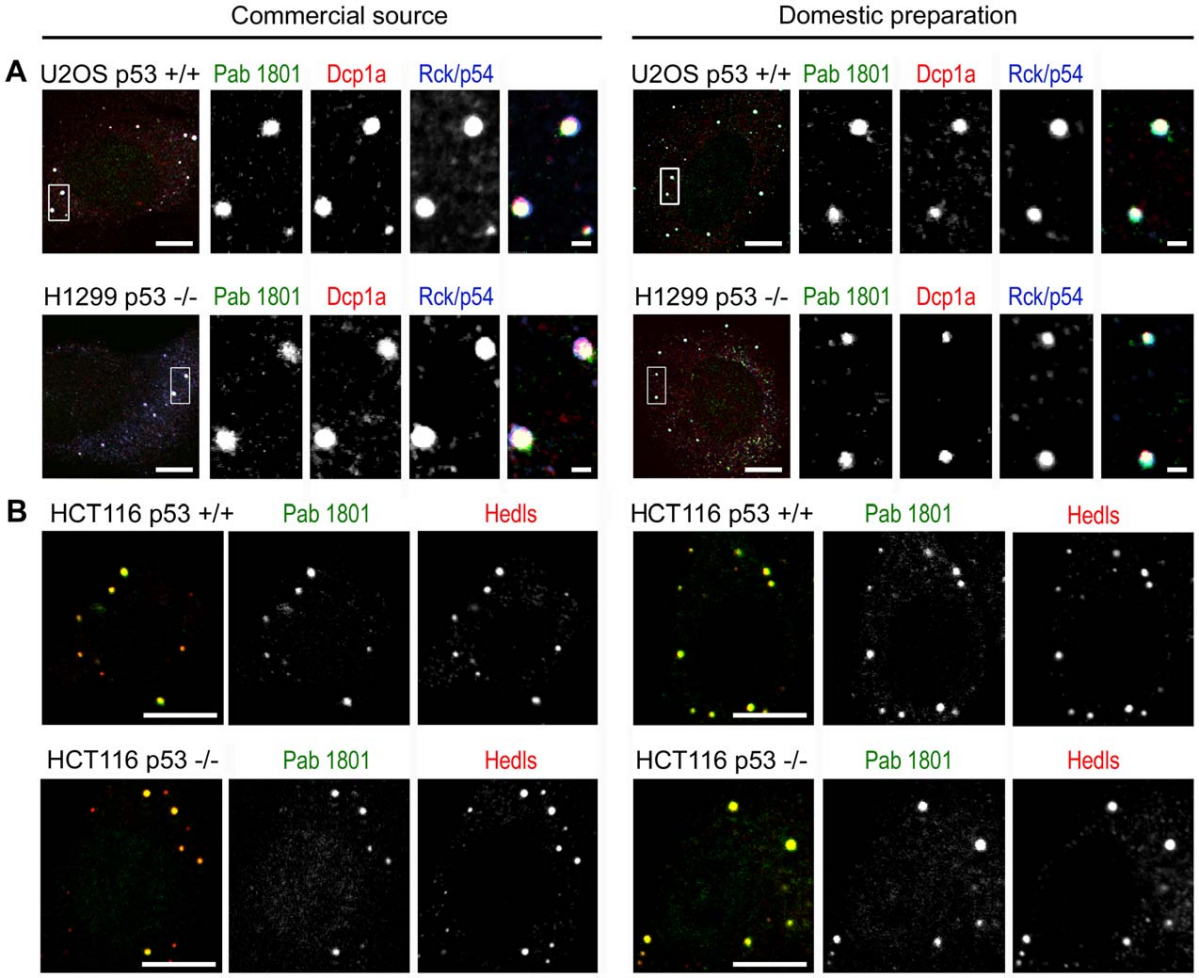
Commercial and domestic sources of Pab 1801 behave similarly. A, U2OS p53+/+ and H1299 p53−/− were simultaneously stained with Dpc1a, Rck/p54 and Pab 1801 from a commercial source (left panels) or from domestic preparations of hybridome supernantant (right panels). B, HCT 116 p53+/+ and HCT116 p53−/− were simultaneously stained with Hedls and Pab 1801 from the same two sources. The strong pattern of Pab 1801 puncta was similar for both commercial and domestic sources of the Pab 1801 in p53+/+ and p53−/− cell lines. Bars, whole cells, 10 µm; magnifications, 1 µm.

Next, we assessed whether distinct preparations of Pab 1801 from other sources behaved similarly. We stained two p53-positive cell lines and two p53 -negative cell lines with Pab 1801 from a commercial source (see Materials and Methods), and found that this antibody preparation also stained cytoplasmic puncta. As with the domestic preparation, the Pab 1801 puncta coincided with PBs stained with Dcp1a, Rck/p54 and Hedls. In summary, two independent preparations of Pab 1801 showed a strong staining of PBs in both p53+/+ and −/− cells (Figure 8 A and B).

**Figure 9:**
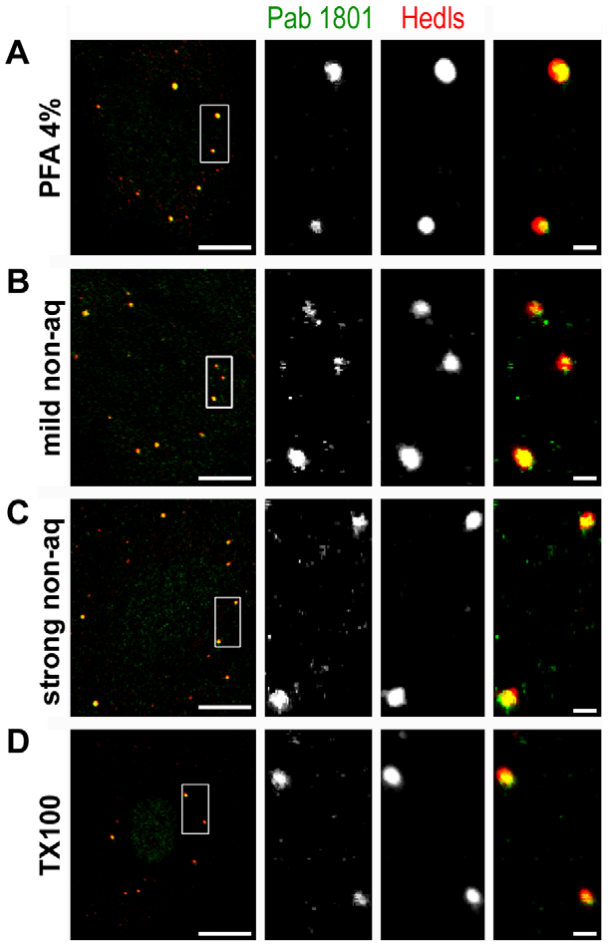
Different procedures give the same immunostaining pattern of Pab 1801. U2OS cells were treated as indicated and immunostained with Pab 1801 and Hedls. A, U2OS cells were fixed as usual in 4% PFA. B, live U2OS cells were treated with methanol-acetone during 3 minutes at room temperature (mild non-aq). C, live U2OS cells were treated with methanol-acetone during 20 minutes at −20°C (strong non-aq). D, live U2OS cells were permeabilized with 0.1% Triton X-100 for 10 minutes prior to fixation in PFA 4%. Nuclear staining with Pab 1801 looks stronger than in control cells. None of these treatments affected the cross-reaction of the Pab 1801 with PBs. Bars, whole cells, 10 µm; magnifications, 1 µm.

**Figure 10:**
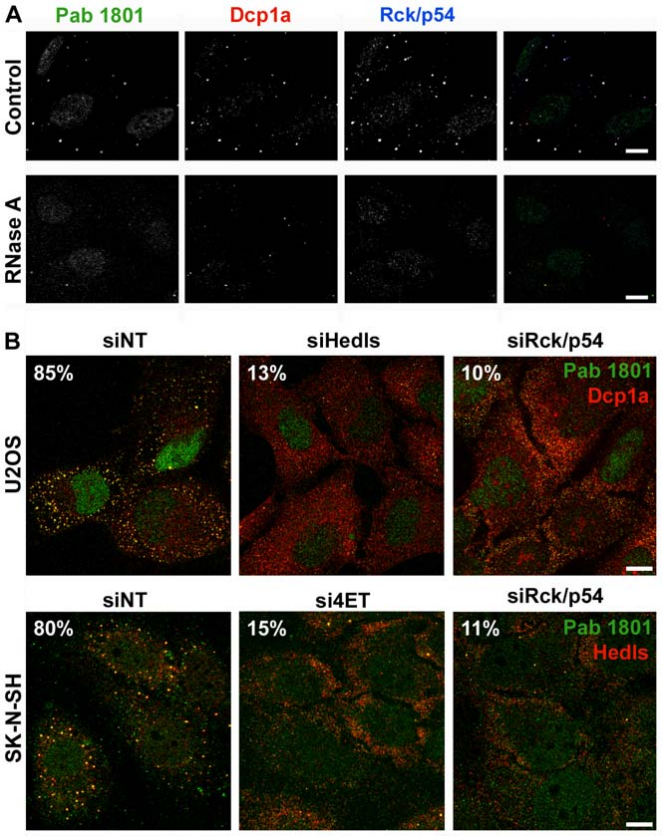
RNase treatment or knockdown of specific PB components provokes the simultaneous dissolution of PBs and Pab 1801 puncta. A, live U2OS cells were permeabilized with 0.1% TX100 for five minutes and then exposed to CSKB without (Control) or with 100 µg/ml of RNase (RNase) for additional 5 minutes. The percentage of cells with PBs simultaneous stained with Dcp1a and Pab 1801 was evaluated in 100 cells from duplicate experiments for each treatment and diminished from 75% in control cells to 40% in RNAse-treated cells. The remaining Pab 1801 foci have less than half of the size of control cells. B, U2OS and SK-N-SH cells were treated with specific siRNAs against the PB components Hedls, Rck/p54 or 4ET, and stained with the indicated antibodies. The Pab 1801 puncta vanished when PB are disrupted. The proportion of cells with *foci* is indicated, as evaluated in 100 cells from duplicate experiments for each treatment. As before (Figures 4, 5 and 6), all puncta were double stained for each pair of antibodies and single-stained *foci* were not present in any of the treatments. Bar, 10 µm.

Finally, we investigated whether distinct procedures affect the outcome of the immunostaining. We treated live U2OS cells with methanol-acetone, a non-aqueous fixation previously reported to yield cytoplasmic puncta upon 1801 staining [6], [7], as indicated in materials and methods. We found that the Pab 1801 also stained PBs upon this procedure (Figure 9B). A stronger fixation with methanol-acetone at −20°C also rendered preparations where PBs were strongly recognized by the Pab 1801 (Figure 9C). Finally, we assessed a procedure known to eliminate most cytosolic components, which is based on treatment of live cells with non-ionic detergents [22], [29]. We treated U2OS cells with 0.1% Triton X-100 during 5 or 10 minutes prior to fixation with PFA 4%, as indicated in materials and methods. We found that this treatment did not affect the cross-reaction of the Pab1801 with PBs (Figure 9D, TX100 and Figure 10A, control). Collectively, these observations indicate that the Pab 1801 stains PBs in several cell types under a variety of conditions.

### The Knockdown of Structural PB Components Provoked the Dissolution of the Pab 1801 Puncta

To further demonstrate the cross-reactivity of the 1801 antibody with PBs, we challenged PB integrity by several strategies. First, we assessed the effect of RNase digestion, a treatment that dissolves PBs [30]. We treated U2OS cells as indicated in materials and methods and, as expected, we found that RNAse treatment greatly reduced the number of cells with PBs from 75% to 40%. The Pab 1801 dotted signal was similarly affected (Figure 10A). In addition, the size of the remaining *foci* was reduced to less than half in comparison with that in control cells (Figure 10A).

Next, PB integrity was challenged by knocking down specific molecules required for PB assembly. We treated U2OS and SK-N-SH cells with small interfering RNA (siRNA) against Hedls, Rck/p54 or 4ET (eIF4E-Transporter), as previously described [23], [27]. The knockdown of these molecules was monitored by western blotting or RT-PCR. In all cases, expression levels was reduced to less than 15% relative to control values (not shown, see also ref [27]). Then, we visualized PBs by staining of Dcp1a or Hedls (Figure 10B). As reported before [23], [27], we found a significant reduction in the number of cells containing visible PBs after knockdown of each one of these PB components. We found that 80 to 85% of the cells treated with the non-targeting siRNA (siNT) contained PBs, and this value was reduced to less than 15% upon knockdown of Hedls, Rck/p54 or 4ET (Figure 10B). Strikingly, paralleling PB disappearance, the dotted cytoplasmic Pab 1801 signal vanished upon knockdown of each one of these PB components. In all cases, the Pab 1801 puncta were detected only when PBs were visible, and showed strict colocalization with Dcp1a in both U2OS and SK-N-SH cells (Figure 10B). Collectively, these results indicate that the Pab 1801-positive puncta colocalize with PBs identified by several markers, and vanished when PBs are no longer present.

PBs contain hundreds of non-related molecules, most of them RNA-binding proteins involved in different functions [11], [20]. In western blot assays, we found that the Pab 1801 antibody recognizes multiple bands in addition to p53 (Figure S1). These bands of around 75, 60, 36, 33 and 30 kDa were present in two p53-negative cell lines, and in their corresponding p53-positive counterparts. We were unable to isolate these proteins by immunoprecipitation, and therefore, whether they represent the PB component recognized by the Pab 1801 remains unknown.

**Figure 11:**
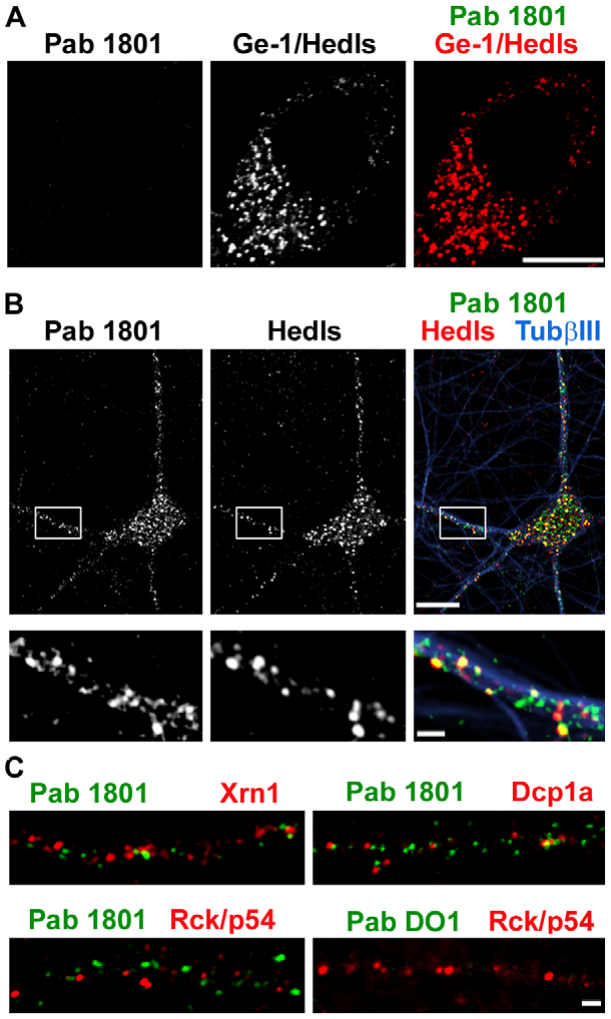
The Pab 1801 does not recognize *Drosophila* PBs, and stains a subset of PBs in rat neurons. A, S2R+ cells were immunostained with the Pab 1801 and an antibody against Ge-1/Hedls. No signal of the Pab 1801 was observed. Bars, 10 µm. B, rat primary neurons were simultaneously stained with Pab 1801, Hedls and Tubulin β III. Pab 1801 puncta and Hedls colocalize partially. Bars, whole neurons, 10 µm; dendrites, 1 µm. C, representative dendritic fragments showing immunostaining against Pab 1801 and the PB components Xrn1, Dcp1a and Rck/p54. As above, Pab DO1 yielded no signal. Bars, 1 µm.

Having demonstrated that the Pab 1801 recognize cytoplasmic puncta in rat neurons (Figure 3) – an organism were the Pab 1801 does not recognize p53 – we assessed the cross-reactivity of the Pab 1801 with PBs in *Drosophila* cells. We used S2R+ cells, where PBs can be visualized by the same markers as in mammalian cells [11], [29], [31]. We found that *Drosophila* PBs, recognized by Ge-1/Hedls were not recognized by Pab 1801, which did not reveal any cytoplasmic puncta (Figure 11A). This indicates that the PB component(s) recognized by the Pab 1801 is not conserved in flies.

PBs are present in all cell types, but in contrast to cell lines, where PBs are quite homogenous, a number of different PBs with distinct composition were reported to be present in neurons, particularly in dendrites (reviewed in [11]). Given that the Pab 1801 puncta are detected in cultured rat neurons (Figure 3), we used these cells to interrogate which components are present in the PBs recognized by the Pab 1801. We found that 38% of the Pab 1801 puncta colocalize with PBs containing Hedls (Figure 11B), suggesting that a component of these PBs, but unlikely Hedls, is cross-reacting with the Pab 1801. Xrn1 label a distinct subset of neuronal PBs [32], and we found that they are not stained by the Pab 1801 (Figure 11C). In neurons, Dcp1a and Rck/p54 label a distinct set of PBs [32], and we found that the Pab 1801 puncta did not colocalize with any of these two markers (Figure 11C). As above, the Pab DO1 yielded no signal above background staining (Fig 11C).

While the identification of the cross-reactive protein(s) will demand intensive work, we have so far ruled out cross-reactivity of the Pab 1801 with a number of PB components, namely Dcp1a, Dcp1b, Rck/p54, Dcp2, and 4ET by means of a transient-transfection approach (see supporting information S1, supporting materials and methods S1 and figure S2).

Altogether, these observations indicate that Pab 1801 recognize a PB component different from Dcp1a; Dcp1b; Dcp2, Rck/p54, Xrn1, 4ET and Hedls, and that is conserved in human and rodents, but not in *Drosophila.*

## Discussion

We have combined pharmacological and siRNA approaches with imaging analysis to reveal a previously unknown cross-reaction of the Pab 1801 with PB component(s). Strikingly, these Pab 1801-positive cytoplasmic puncta do not contain p53, since they are also present in two independent p53-negative cell lines. In addition, Pab 1801-cytoplasmic puncta were observed in rat cells, despite the fact that rat p53 lacks the Pab 1801-specific epitope [18]. In contrast, the Pab 1801 nuclear staining corresponds to genuine p53, as it is upregulated by p53-activating stimuli and absent in p53-null cells.

We also demonstrated that the Pab 1801 puncta are the result of a cross-reaction of this monoclonal antibody with a yet unidentified PB component, This crossreactivity was observed upon a variety of immunostaining strategies, including procedures previously used by several authors to show the presence of p53 cytoplasmic dots in neuroblastoma cells [6], [7].While the cross-reacting molecule remains to be determined, we have shown that its aggregation in PBs is modulated similarly to most PB components, as pharmacological or siRNA-mediated PB disruption dramatically affects the detection of Pab 1801 puncta. This elusive molecule that shares an epitope with human p53 is conserved in human and rodents, and our studies suggest that is distinct from Dcp1a, Dcp1b, Dcp2, Rck/p54, Xrn1, 4ET and Hedls. PBs are complex supramolecular aggregates of RNPs, and their protein composition is not completely known. Currently, hundreds of proteins with different functions are known to be recruited to PBs [11], [20], and novel PB components are expected to be identified with the help of high performance approaches that are underway. This information will be highly valuable for the identification of the PB component(s) recognized by Pab 1801.

The Pab 1801 monoclonal antibody was raised against a recombinant polypeptide including amino acids 32 to 393 of human p53, and the epitope spans from amino acids 32 and 79 [18]. Later on, Soussi and col. [19] mapped the epitope to a fragment from amino acid 46 to 55, with the sequence SPDDIEQWFT. A survey of the most important PB components indicates that no significant homology occurs between this 10 amino acid sequence and Dcp1a, Dcp1b, Dcp2, Hedls, Rck/p54, 4ET, Xrn1, GW182, or Lsm1. However, protein conformation may affect antibody binding, and the epitope may be split in more than one peptide segments. Interestingly, the PB component Edc3 includes a conserved 4 amino acid sequence (DDIE), which is not present in Drosophila Edc3 and that may potentially be recognized in the context of the full molecule, where additional contact sites would be provided by amino acids somewhere else in the molecule. This remains to be investigated. In contrast, post-translational modifications are unlikely to be involved in the cross-reactivity reported here, given that the immunogen used for the preparation of this monoclonal antibody was a recombinant polypeptide.

The cytoplasmic localization of p53 is an emerging aspect in the cell biology of this important transcriptional regulator [2]. Having reliable tools is critical to assess p53 localization to the mitochondria, cytosol or cytosolic aggregates, three major cytoplasmic compartments where p53 is located upon different conditions, and with different consequences in cell homeostasis [1], [2]. The cross-reactivity of the Pab 1801 antibody with PBs was previously overlooked, and this antibody was used to conclude the occurrence of p53 cytosolic aggregates under a variety of conditions [6], [7]. Our observations imply that imaging of cytoplasmic p53 with the Pab 1801 requires careful interpretation and controls.

## Materials and Methods

### Ethic Statement

All the work involving animals was performed at Instituto Leloir where NIH regulations are followed at the animal facility (Assurance number A5168-01). The protocol was specifically approved by the Institutional Animal Care and Use Committee of the Fundación Instituto Leloir (IACUC-FIL, Protocol 2009 08 26GB) according to the Principles for Biomedical Research involving animals of the Council for International Organizations for Medical Sciences and provisions stated in the Guide for the Care and Use of Laboratory Animals.

### Cells and Drug Treatments

The human osteosarcoma cell line U2OS, the human fibroblast-like fetal lung cell line WI 38, the human neuroblastoma SK-N-SH cell line, the human non-small cell lung carcinoma cell line H1299 were from the ATCC (American Tissue Culture Collection). The human colonic epithelial HCT116 p53+/+ and −/− cells were originally obtained from Bert Volgestein laboratory [16]. All cell lines were maintained in DMEN or McCoy medium supplemented with 10% fetal bovine serum (Natocor, Córdoba, Argentina), penicillin and streptomycin (Sigma). Schneider S2R+ cells from the *Drosophila* Genomic Resource Center (Indiana University, Bloomington, IN) were grown in M3+ BYPE supplemented as above [29], [31]. Hippocampal cultures were prepared as described previously [27]. Briefly, hippocampi were dissected from Sprague Dawley rats at embryonic day 18 and digested with trypsin. Cells were seeded on poly-d-lysine (Sigma-Aldrich)-coated glass coverslips. Cultures were maintained in Neurobasal medium (NB; Invitrogen) supplemented with B27 (Invitrogen) and glutamine (complete NB; Invitrogen) at 5% CO_2_.

When using chemicals, stock solutions were always diluted into conditioned medium. The final concentrations of the compounds used were: thapsigargin, 1 µM (SIGMA); cycloheximide, 250 µg/ml (SIGMA); puromycin, 100 and 250 µg/ml (SIGMA); arsenite, 250 and 500 µM (SIGMA); daunorubicin, 0.22 µM (Oncogene Research Products); actinomycin D, 5 nM (Calbiochem).

### Plasmids, Cell Transfection and Immunofluorescence

Smaug1-ECFP construct was previously described [26], [27]. Cell lines were transfected with either Lipofectamine 2000 Transfection Reagent (Invitrogen) or Jet Prime (Polyplus Transfection) following manufacturer’s instructions. Immunofluorescence was performed as previously described [22], [23], [29] Mild non-aqueous fixation with methanol-acetone 1∶1 was performed 3 minutes at room temperature [6] and strong non-aqueous fixation was done with the same solution for 20 minutes at −20°C. In both types of treatments immunofluorescence was done without permeabilization. Permeabilization of live cells with 0.1% of the non-ionic detergent Triton X-100 in CSKB (CSK buffer) was performed during 5 and 10 minutes prior to fixation in PFA 4% [22], [29]. When indicated, treatment with RNase A (Sigma) (100 µg/ml in CSKB) was performed for 5 minutes after 5 minutes of live permeabilization with 0.1% TX100 in CSKB.

Monoclonal Pab 1801 was obtained by immunization with a recombinant peptide of amino acids 32 to 393 of human p53 [18]. Two different sources were used: hybridome’s supernatant without dilution was used in figures 1, 2, 3, 4, 5, 6, 7, 8, 9, 10, 11 and commercial Pab 1801 diluted 1∶100 (sc-98, Santa Cruz, CA, USA) was used in figure 8 and figure S1. The rest of monoclonal anti p53 antibodies derived from hybridome’s supernatant and used without dilution were the following: Pab DO1, prepared using recombinant wt human p53 (recognizes amino acids 11 to 25) [33]. Pab 240, generated against a fusion protein containing amino acids 156–214 of mammalian p53 [34]. Pab 421, raised against mouse p53 protein (recognizes amino acids 371–380) [35]. Primary antibodies were diluted as follows: polyclonal rabbit anti-p53 FL 393 (Santa Cruz, CA, USA), 1∶100; monoclonal IgG2a anti Dcp1a (Abnova Corporation, Taiwan), 1∶1000; polyclonal rabbit anti-Hedls and anti Rck/p54 (Bethyl; TX, USA), 1∶500; polyclonal anti-Staufen [22], 1∶400; goat anti-TIA1 (Santa Cruz, CA, USA), 1∶100. Secondary antibodies coupled to Alexa 488, Alexa 555 or Alexa 666, used at 1∶500–1∶1000, were from Molecular Probes (Invitrogen Corporation, Carlsbad CA, USA); or coupled to Cy2, Cy3 or Cy5, used at 1∶300–1∶500, were from Jackson ImmunoResearch Laboratories (West Grove, PA, USA). In all the immunostainings were monoclonal Pab 1801 (IgG1) was combined with monoclonal anti Dcp1a (IgG2a), anti isotypes coupled to A 488 or A555 from Molecular Probes (Invitrogen Corporation, Carlsbad CA, USA) were used at 1∶500 (Figures 4, 5, 7A, 8, 10A and B, 11B and C).

### siRNA-treatment

U2OS cells were treated with a non-targeting siRNA (siNT) 5′-UAGCGACUAAACACAUCAA, a siRNA anti-Hedls (siHedls, Cat. Num. 004397, Dharmacon); anti-Rck/p54 (siRck/p54, Cat. Num. 0143295, Dharmacon); or against 4ET (5′GAACAAGAUUAUCGACCUA) [36] used at 50 nM during 72 hs. Jet Prime (Polyplus Transfection) was used as transfection reagent and manufacturer instructions were followed.

### Imaging

Images were acquired in a PASCAL-LSM or a LSM510 Meta confocal microscopes (Carl Zeiss, Oberkochen, Germany), using C-Apochromat 40×/1.2 W Corr or 63×/1.2 W Corr - water immersion objectives for the LSM, and a EC “Plan-Neofluor” 40×/1.30 Oil or Plan-Apochromat 63×/1,4 Oil for the LSM510 Meta. Images were acquired with Zeiss LSM software, and pixel intensity was always lower that 250, being 255 saturating levels. Equipment adjustment was assessed by using 1 µm FocalCheck fluorescent microspheres (Molecular Probes). No filters or gamma adjustment were used previous to the analysis of number of cells with foci or foci size using the ImageJ software.

### Western Blot

Western blotting was performed by standard procedures using PVDF membranes (Immobilon-P polyvinylidene difluoride, Millipore, Bedford, MA), LumiGlo (Cell Signaling) and Hyperfilm (Amersham Biosciences). Hybridome supernatant from Pab DO1 was used diluted in 5% milk/PBST. Anti β-actin (SIGMA) was diluted 1∶5,000. For semi-quantitative analysis, autoradiographs were scanned and signal intensity evaluated with the ImageJ software.

## Supporting Information

Figure S1 **Western blot with the Pab 1801 gives multiple bands.** Whole lysates from U2OS, H1299, HCT166 p53+/+ and HCT p53−/− cells were immunoblotted with commercial and domestic Pab 1801. p53 is present in p53+/+ cells but not in p53−/− cells. Additional bands at around 75, 60, 36, 33 and 30 kDa were detected.
(TIF)

Figure S2 **The Pab 1801 does not cross react with Dcp1a, Dcp1b, Rck/p54 or Dcp2.** U2OS cells were transfected with human Dcp1a (A); Dcp1b (B); Rck/p54 (C) or Dcp2 (D) fused to FLAG, and stained with an antibody against FLAG (see supplementary Material and Methods). Simultaneously, cells were stained with the Pab 1801. Representative transfected cells for each construct are shown in the left panels, and examples of neighbouring non-transfected cells for each case are shown in the right. None of the overexpressed PB components increased the signal intensity of the Pab 1801 staining. Bars, 10 µm.
(TIF)

Supporting Information S1 **Supporting information corresponding to transient-transfection approach to ruled out cross-reactivity of the Pab 1801 with PB components Dcp1a, Dcp1b, Rck/p54, Dcp2, and 4ET** [**37**] **.**
(DOCX)

Supporting Materials and Methods S1 **Supporting materials and methods corresponding to transient-transfection of the PB components Dcp1a, Dcp1b, Rck/p54, Dcp2, and 4ET** [**37**] **.**
(DOCX)
