# Supplementary figures and images for: A Monoclonal Antibody against p53 Cross-Reacts with Processing Bodies
Source: PLoS One. 2012 May 10;7(5):e36447. doi: 10.1371/journal.pone.0036447 (PMC3349707; doi:10.1371/journal.pone.0036447)

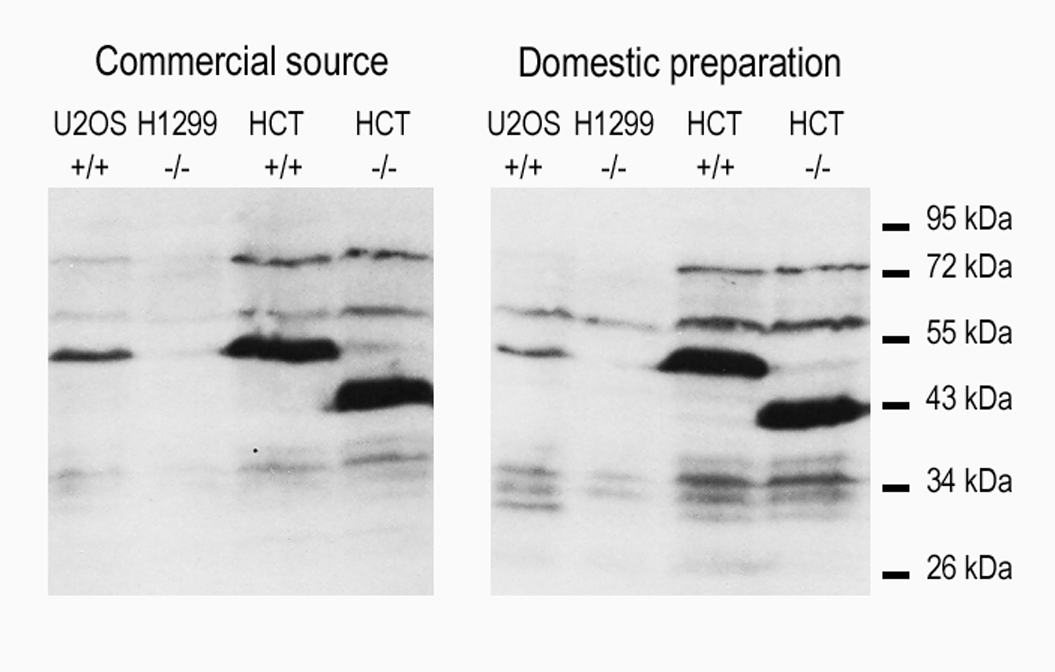

Supplement: Figure S1 — Western blot with the Pab 1801 gives multiple bands. Whole lysates from U2OS, H1299, HCT166 p53+/+ and HCT p53−/− cells were immunoblotted with commercial and domestic Pab 1801. p53 is present in p53+/+ cells but not in p53−/− cells. Additional bands at around 75, 60, 36, 33 and 30 kDa were detected. (TIF) [file pone.0036447.s001.tif]

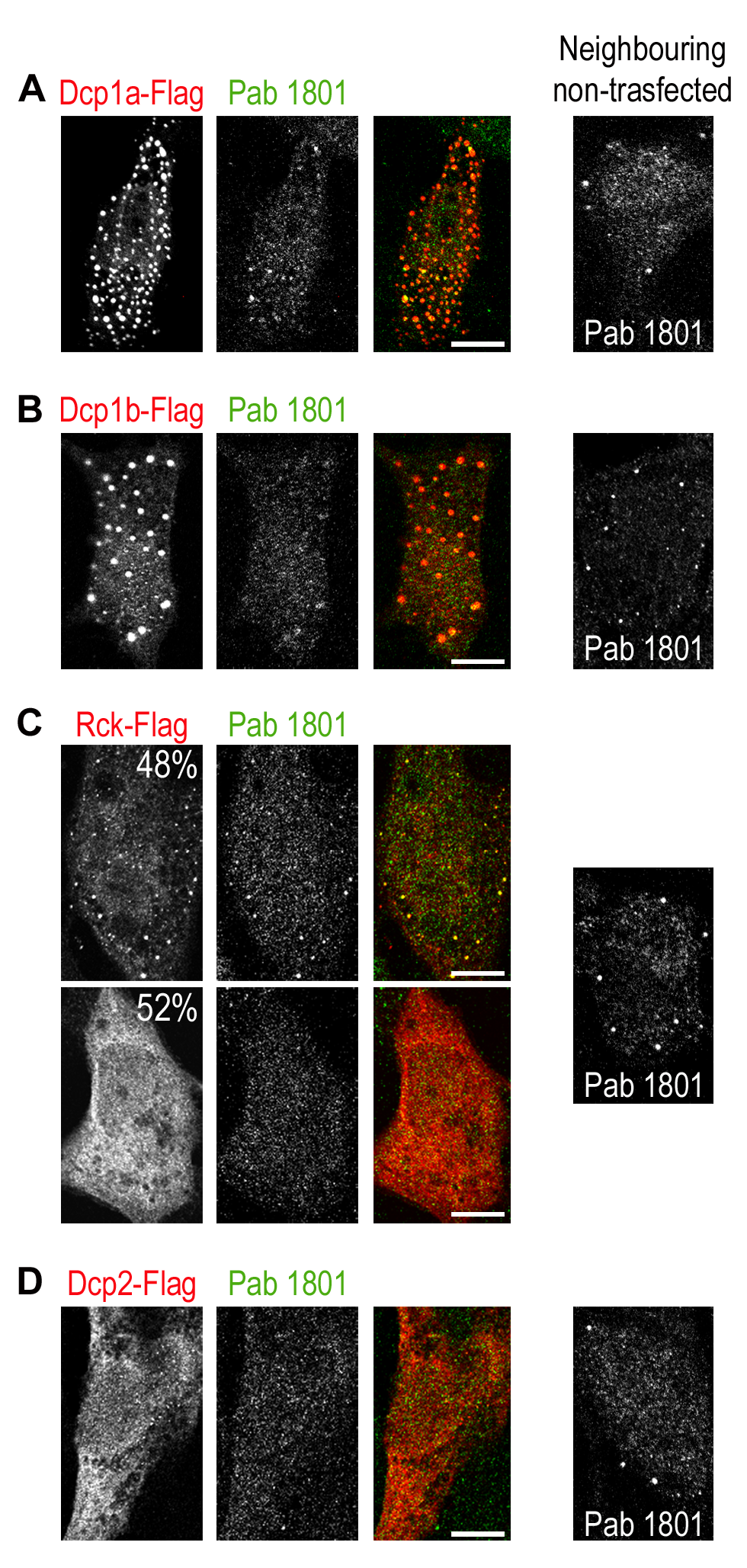

Supplement: Figure S2 — The Pab 1801 does not cross react with Dcp1a, Dcp1b, Rck/p54 or Dcp2. U2OS cells were transfected with human Dcp1a (A); Dcp1b (B); Rck/p54 (C) or Dcp2 (D) fused to FLAG, and stained with an antibody against FLAG (see supplementary Material and Methods). Simultaneously, cells were stained with the Pab 1801. Representative transfected cells for each construct are shown in the left panels, and examples of neighbouring non-transfected cells for each case are shown in the right. None of the overexpressed PB components increased the signal intensity of the Pab 1801 staining. Bars, 10 µm. (TIF) [file pone.0036447.s002.tif]
