# Supplementary material for: A Monoclonal Antibody against p53 Cross-Reacts with Processing Bodies
Source: PLoS One. 2012 May 10;7(5):e36447. doi: 10.1371/journal.pone.0036447 (PMC3349707; doi:10.1371/journal.pone.0036447)
Supplement: Supporting Information S1 — Supporting information corresponding to transient-transfection approach to ruled out cross-reactivity of the Pab 1801 with PB components Dcp1a, Dcp1b, Rck/p54, Dcp2, and 4ET [37] . (DOCX) [file pone.0036447.s003.docx]

**SUPPORTING INFORMATION**

We transfected U2OS cells with cDNAs encoding FLAG-tagged Dcp1a, Dcp1b, Rck/p54, Dcp2, or 4ET, which were detected with an anti-FLAG antibody, used simultaneously with the Pab 1801 against p53. If the transfected molecule were recognized by the Pab 1801, an increase in the Pab 1801 staining should be expected upon overexpression. This was not the case for any of the constructs assessed. In agreement with previous reports [[37](#_ENREF_37)], we observed that Dcp1a and Dcp1b formed numerous and conspicuous *foci* in all transfected cells. We found that these *foci* were stained by the Pab 1801, with similar or lower intensity than the endogenous PBs present in non-transfected cells (Figure S2A, B, compare transfected and neighbouring non-transfected cells). As previously described, Rck/p54 formed large cytoplasmic aggregates in half of the transfected cells. We found that the signal of Pab 1801 was not proportionally increased in these large *foci*, which were weakly stained by the Pab 1801 (Figure S2C, compare transfected and neighbouring non-transfected cells). In the remaining transfected cells, overexpressed Rck/p54 appeared soluble, and PBs dissolved, as reported before [[37](#_ENREF_37)]. In these cells, the Pab 1801-puncta were no longer detected and, remarkably, soluble Rck/p54 was not recognized by the Pab 1801, as the intensity of Pab 1801 staining was comparable in overexpressing and non-expressing cells. As reported before [[37](#_ENREF_37)], a similar dissolving effect on PBs was observed upon Dcp2 overexpression. In addition, we found that the Pab 1801 cytoplasmic puncta were absent in Dcp2-transfected cells and that the overall Pab 1801 cytoplasmic signal did not increase. These observations indicate that the Pab 1801 does not recognize soluble overexpressed Dcp2 (Figure S2D). Finally, overexpression of 4ET did not increase the immunofluorescence signal of the Pab 1801 (unpublished data). Collectively, these observations suggest that none of the assessed PB molecules –namely Dcp1a; Dcp1b; Rck/p54; Dcp2 and 4ET- cross-reacts with the Pab 1801.

This is in accordance with the observations described in the main text (Figure 11).
