# Supplementary material for: A Monoclonal Antibody against p53 Cross-Reacts with Processing Bodies
Source: PLoS One. 2012 May 10;7(5):e36447. doi: 10.1371/journal.pone.0036447 (PMC3349707; doi:10.1371/journal.pone.0036447)
Supplement: Supporting Materials and Methods S1 — Supporting materials and methods corresponding to transient-transfection of the PB components Dcp1a, Dcp1b, Rck/p54, Dcp2, and 4ET [37] . (DOCX) [file pone.0036447.s004.docx]

**SUPPORTING MATERIALS AND METHODS**

Western blot was done as described in Materials and Methods. Commercial Pab 1801 (Santa Cruz, CA, USA) was diluted 1:500. Hybridome supernatant from Pab 1801 was used diluted 1/2 in 5% milk/PBST.

hDcp1a-Flag, hDcp1b-Flag, hDcp2-Flag and hRck-Flag were kindly provided by Dr. Jens Lykke –Andersen [[37](#_ENREF_37)]. Cell transfection and immunofluorescence were done as described in materials and methods. Goat Anti-FLAG (Abcam, UK) was used 1:500.
